# Clinical Manifestations and Laboratory Findings of Kawasaki Disease: Beyond the Classic Diagnostic Features

**DOI:** 10.3390/medicina58060734

**Published:** 2022-05-30

**Authors:** Wendy Lee, Chooi San Cheah, Siti Aisyah Suhaini, Abdullah Harith Azidin, Mohammad Shukri Khoo, Noor Akmal Shareela Ismail, Adli Ali

**Affiliations:** 1 Department of Pediatric, Faculty of Medicine, Universiti Kebangsaan Malaysia, Jalan Yaacob Latif, Kuala Lumpur 56000, Malaysia; a172052@siswa.ukm.edu.my (W.L.); a171637@siswa.ukm.edu.my (C.S.C.); a167541@siswa.ukm.edu.my (S.A.S.); a167553@siswa.ukm.edu.my (A.H.A.); 2 Department of Pediatric, Hospital Wanita dan Kanak Kanak Sabah, Kota Kinabalu 88996, Malaysia; shukrikhoo@moh.gov.my; 3 Department of Biochemistry, Faculty of Medicine, Universiti Kebangsaan Malaysia, Jalan Yaacob Latif, Kuala Lumpur 56000, Malaysia; nasismail@ukm.edu.my

**Keywords:** Kawasaki disease, intravenous immunoglobulin, coronary artery aneurysm

## Abstract

Kawasaki disease (KD) has shown a marked increase in trend over the globe, especially within the last two decades. Kawasaki disease is often seen in the paediatric population below five years old, while it is rare for those who are beyond that age. Up to this date, no exact causes has been identified although KD was found more than half a century ago. The underlying pathogenesis of the disease is still unelucidated, and researchers are trying to unlock the mystery of KD. To further complicate the diagnosis and the prompt management, a specific biomarker for the diagnosis of KD is yet to be discovered, making it hard to differentiate between KD and other diseases with a similar presentation. Nonetheless, since its discovery, clinicians and scientists alike had known more about the different clinical aspects of typical KD. Thus, this article intends to revisit and review the various clinical manifestations and laboratory characteristics of KD in order to guide the diagnosis of KD.

## 1. Introduction

Kawasaki disease is a systemic idiopathic febrile vasculitis that often affects children under 5 years [1]. In Japan back in 1961, KD was initially reported and subsequently recognised based on the profiling of 50 patients with a similar phenotype by Dr Tomisaku Kawasaki in 1967 [2]. Kawasaki disease was then identified as the most common cause of acquired heart disease in paediatric populations as it would subsequently result in coronary artery aneurysms if left untreated [1,2].

Although KD has been reported in an increasing trend worldwide, the aetiology of KD remained a mystery. Various aetiology has been proposed such as genetic [3], bacterial and viral infection including rotavirus, adenovirus, enterovirus and coronavirus [4], exposure of maternal smoking [5], ethnicity [6], and a strong family history [7] of KD, thus making the explanation of KD pathogenesis more complicated. According to the American Heart Association, KD is diagnosed clinically which further classified into typical and atypical KD. However, often KD is being misdiagnosed as measles, non-exudative pharyngitis, retropharyngeal abscess, bacterial cervical adenitis and scarlet fever due to their similar manifestations with KD [1,8,9,10]. Additionally, the multi-system involvement in KD contributes to the challenges in diagnosing KD. Despite the complexity of KD, the mainstay treatment has been proven effective. Prompt treatment of intravenous immunoglobulin (IVIG) in the acute phase of KD has been shown to reduce the occurrence of coronary artery abnormalities [11,12]. A high dosage of aspirin is commonly given along with IVIG for KD for years due to its anti-inflammatory property [13]. Various alternatives or adjuncts including corticosteroid, infliximab, etanercept, plasmapheresis as well as administering a secondary IVIG infusion among KD patients who are found to be IVIG resistant [1]. The general outcome of KD is favourable if a correct and timely clinical diagnosis is made, allowing a prompt and specific treatment to be administered. Thus, this article will concisely review and update: (i) the current changes in the epidemiology of KD, (ii) the myriad clinical manifestations of KD, including cardiac lesions and (iii) the associated laboratory investigations, which will assist clinicians in establishing the diagnosis and managing patients with KD.

## 2. The Global Epidemiology of KD

Kawasaki disease, which occurs predominantly among infants and children, has slowly emerged to become a global disease as it has been identified in more than 60 countries including Asia, the Middle East, Latin America, Africa, North America, and Europe [6]. Among these, the prevalence of KD was noticeably high among East Asian countries such as Japan, Taiwan, and Korea. Undeniably, the incidence of KD in Japan was the highest in which the cases had steadily increased from 1970 to 2018 [7,11,12,13,14,15]. In contrast, Southeast Asian countries, namely Malaysia and Singapore, have seen less significant rates of KD in comparison to the top three East Asian countries mentioned earlier [16,17,18,19]. Nonetheless, in Thailand, it was reported that there was an increasing incidence rate of KD from 1998 to 2002 among children aged 0 to 2 years old [6,20]. China, which is one of the major Asian countries, had reported several data based on different provinces including Shanghai, Yun Nan, Si Chuan, Bei Jing, Guang Dong, Jiang Su and Shanxi [21,22,23]. Kawasaki disease has also been reported by the Middle East countries, including Turkey and Iran, however, the number of cases of KD reported is inconclusive due to the lack of data from children below the age of 5 from the national registry. Interestingly, Egypt also had reported one case of a misdiagnosed KD as myocardial infarction, suggesting that misdiagnosed KD might be the plausible reason why the cases were under-reported [24,25].

A similar trend was also seen outside of the Northeast Asian countries. The incidence of KD reported by Uehara et al. in the United States was 19.1 per 100,000 among children less than 5 years old, while in Canada, the figure was 19.6 per 100,000 in children less than 5 years old [26]. In England, the prevalence of KD was 8.39 per 100,000 children [27] and the prevalence of 9.34 per 100,000 children was reported among children under 5 years in Australia [28]. As we compare the incidences of Northeast Asian and outside of Northeast Asian region countries, the incidence was without any significant elevation or reduction. In a larger overview, the incidences of KD in the Northeast Asia region countries, including Taiwan, Korea, Japan, and China are 10–30 times more than in the US and Europe [29]. A similar trend also revealed that KD was reported higher among the children of Asian-Pacific Islander descent, when compared to other ethnicities in the United States. This can be seen through the number of reported cases among American Japanese children in Hawaii, which was similar to the incidence observed in Japan. The data has suggested that, although in a general overview, the non-Asian countries documented a lower incidence rate of KD when compared to the Northeast Asian countries, we must further scrutinise the data based on ethnicity, as it is one of the important determinants of the incidence of KD [6,30,31]. However, some countries may have under-reported KD due to misdiagnosed cases and/or inadequate medical facilities and experts, thus requiring more efforts and a better disease understanding for proper diagnosis of KD. Therefore, it is crucial to determine a more robust tool to diagnose KD, which can be used to later determine and reflect the true epidemiology of KD worldwide.

## 3. Diagnostic Clinical Features of KD

Kawasaki disease is diagnosed through a constellation of classic clinical features, which have remained in use and was hardly changed since its first description 50 years ago. Based on the current guideline by McCrindle et al., patients with a high-grade fever (more than 39 degrees Celsius) for at least 5 days; coupled with at least 4 out of 5 classic features are diagnosed with Complete KD (Typical KD), while those who do not fulfil the mentioned clinical criteria are categorised as incomplete KD (Atypical KD). The classical features of KD include: (i) erythema and cracking of lips, strawberry tongue, erythema of oral and pharyngeal mucosa; (ii) bilateral bulbar conjunctival injection without exudates; (iii) diffuse maculopapular rash eruptions; (iv) painful erythema and oedema of the hand and feet; (v) unilateral cervical lymphadenopathy. If more than 4 principal clinical criteria are present, especially through redness and swelling of the hands and feet, the diagnosis can be established earlier with only 4 days of fever [1]. This current list of classic criteria remains to be the gold standard of the diagnostic tool for KD.

The presence of fever, although it is originally described to be high grade with a temperature of more than 39 degrees Celcius lasting more than five days; in the real clinical setting, may not always be the case, especially in younger infants [32]. The KD rash is mainly described to be manifested as diffused maculopapular rash eruptions that occur within the onset of a febrile period in the published diagnostic criteria, although various other descriptions of cutaneous manifestations had been reported [1]. The typical KD rashes usually start to become visible on the trunk and start to spread outwards to the extremities as the illness progresses [33]. The other KD skin manifestations may include: (i) diffuse scarlatiniform erythroderma [1] (ii) erythema multiforme-like rashes [34,35] (iii) erythema and oedema of the hand and feet [1,33]. Erythema multiforme usually appears as targetoid lesions, predominantly affecting the torso and extremities [34,35]. During the acute onset of KD, the palms and the soles can appear erythematous, whereas the dorsum of the hand and feet will be oedematous and tender. This will be followed by desquamation which usually starts in the periungual areas 2 to 3 weeks during the subacute phase of KD and subsequently, the appearance of Beau lines during the resolution phase of KD [1,33]. Other KD dermatological features may include: (i) urticarial or fine micro pustular eruptions which mainly involve the trunk and extremities [1]; and (ii) palmoplantar psoriatic eruption with plaques and pustular lesion [1,36,37]. KD associated psoriasiform rashes had been observed to resolve earlier when compared to conventional psoriasis [37]. Interestingly, several studies have also demonstrated a higher risk of developing atopic dermatitis among patients with KD observed during their follow-up [38,39].

Being one of the classic features of KD, cervical lymphadenopathy usually appears unilaterally distributed within the anterior cervical triangle, measuring more than 1.5 cm in diameter [1]. It is very crucial to pay attention that some KD patients may have fever and lymphadenopathy as their sole features. However, there was an insignificant association between patients who presented with only fever as well as lymphadenopathy, with the prognosis of coronary artery aneurysm or IVIG resistance [40]. KD-related lymphadenopathy (LKD) can be distinguished from bacterial cervical lymphadenopathy (BCL) by imaging procedures such as ultrasound or contrast-enhanced computed tomography (CT) [1,41]. LKD usually affects multiple lymph nodes of various sizes, whereas abscesses, phlegmon or a solitary lymph node with a hypoechoic core are more commonly present in BCL [1,42]. The absence of abscesses on CT, at a younger age, and a raised C-reactive protein (CRP) are helpful to differentiate (LKD) from BCL [41]. LKD was found to be affecting more older children and usually presented with more severe inflammatory reactions than KD patients without lymphadenopathy [43], with a higher possibility of developing the life-threatening Kawasaki Shock Syndrome (KDSS) [44].

There was a significant number of reported KD cases presented with severe oral manifestations. These include changes on the lips and in the oral cavity which may be presented as erythema, dryness, fissuring, peeling, vertical cracking, and bleeding of the lips; a strawberry tongue with erythema and prominent fungiform papillae; and diffuse erythema of the oropharyngeal mucosa. This can be seen when the patient’s lips were grossly swollen, and eroded with haemorrhages, leading to marked difficulty to open the mouth [41,45]. In another case, the patient was presented with red and cracked lips, coupled with diffused pharyngeal hyperemia without any exudate resulting in microstomia, which required reconstructive lip surgery with bilateral commissuroplasty [45].

Bilateral nonexudative conjunctivitis is one of the features with a lesser variation in its association with KD, although some reports had documented other ocular manifestations. Bilateral nonexudative conjunctivitis and anterior uveitis are the most common eye manifestations in KD while the involvement of the posterior segment is rare [46]. The manifestation of anterior uveitis in KD is mild and bilateral, sometimes associated with precipitate keratitis [47]. According to a study conducted by Choi et. al, anterior uveitis is a significant ocular sign to help with the diagnosis of Incomplete KD, as the study observed that out of 110 patients with KD, 32 patients presented with anterior uveitis, giving an incidence rate of 29% [48]. It is also noted that the manifestation of anterior uveitis among KD patients is associated with older age groups and the presence of neutrophilia [48]. Other ocular signs that had been associated in patients with KD may include punctuated keratitis, retrodescemetic precipitates, vitritis, bilateral optic disc swelling with papillitis, bilateral iridocyclitis and subconjunctival haemorrhage [48,49,50].

## 4. Other Clinical Manifestations of KD

Interestingly, Kawasaki may also manifest with some additional clinical manifestations, or in some cases with atypical presentation. These manifestations are helpful to provide an additional certainty in the establishment of the diagnosis of KD. These clinical manifestations involve the cardiovascular [1], gastrointestinal [1,17,51], neurological [1,17,52], genitourinary [1,53], musculoskeletal [17,54], and respiratory systems [1,55,56], which are described below.

### 4.1. Cardiac Manifestation of KD

Cardiovascular manifestations are specifically associated with KD and are widely reported all over the world (Table 1). They are recognised as the foremost reason for long-term morbidity and mortality. The cardiovascular abnormalities of KD may include coronary artery abnormalities, aneurysm of medium-sized non-coronary arteries, aortic root enlargement, pericarditis, myocarditis, endocarditis and valvular regurgitation [1]. Non-coronary cardiac features such as mitral regurgitation and aortic root dilation developed in 27% and 8% of the KD patients, respectively [57]. The acute stage of KD manifests pericarditis and myocarditis, while coronary artery aneurysm can only be seen in the second week of the illness [58]. Coronary artery abnormalities in KD include dilation only or aneurysm of various sizes, numbers, and characteristics. Thus, 2-dimensional (2D) echocardiography should be performed in patients suspected of KD. The prevalence of coronary artery lesions detected during the initial echocardiography was 3.6% in Japan [59], with a majority of the affected patients were having coronary artery dilation [29,59,60,61,62,63,64]. The major risk factors of coronary artery lesions may include the incomplete KD diagnosis, IVIG unresponsiveness, and a longer duration of fever [64]. Apart from these, coronary artery lesions were apparentamongst patients who had delayed hospital visits (≥7 days from onset of symptoms) [60]. In Malaysia, about 10% of the patients have developed coronary artery aneurysms and the non-IVIG treated patients (19%) have formed a larger proportion than those who were given IVIG (9%) [17]. This is supported by another study which indicated that around 15% to 25% of the children who were not treated adequately for KD eventually had coronary artery aneurysm or ectasia [65]. The majority of the patients who were diagnosed with coronary artery aneurysms are younger than 5 years old [60,66].

### 4.2. Gastrointestinal System

It is undeniable that some patients with atypical KD are frequently presented with gastrointestinal symptoms which may include abdominal pain and distention, diarrhoea, vomiting, jaundice, gallbladder hydrops, pancreatitis, pseudo-obstruction, paralytic ileus and haematemesis [67,68,69]. Diarrhoea, vomiting, abdominal pain, hepatitis, and gallbladder hydrops are the most frequent gastrointestinal symptoms of KD while pancreatitis and jaundice are less prevalent [1]. 4.6% of the patients with KD that came in with the signs and symptoms of acute abdomen, high fever, and jaundice, mostly suggested the presence of gallbladder hydrops [58]. In Iran, the incidence rate of gastrointestinal presentation in KD patients was 38%, with gallbladder hydrops seen in 1% of those patients [51]. It was far higher when compared to a retrospective study done in Malaysia, where only 6% of KD patients have presented with gastrointestinal symptoms [17]. Kawasaki disease patients with abdominal symptoms have a higher risk of being resistant to IVIG as well as developing coronary aneurysms [69]. Apart from that, the likelihood of patients who are less than one year old to have gastrointestinal symptoms and subsequently coronary artery aneurysm is higher [70]. In terms of pseudo-obstruction, the incidence is unusual to occur [71]. Haematemesis as a clinical presentation of KD is rare and caused by hemorrhagic duodenitis. However, haematemesis can also be seen in other vasculitides, thus diagnosis of KD is often delayed [69].

### 4.3. Genitourinary System

KD is a systemic vasculitis disease. As such, it may involve the kidneys and the urinary tract. The clinical manifestations involving the genitourinary system may include pyuria, prerenal acute kidney injury (AKI), renal AKI caused by tubulointerstitial nephritis (TIN), renal AKI associated with either KD shock syndrome, hemolytic uremic syndrome (HUS), immune-complex mediated nephropathy [72]. Renal abnormalities from ultrasound imaging study TIN will either show normal kidney size or increase in size of kidney as well as hyperechogenicity due to infiltration of the inflammatory cells, as it is the common renal disease in KD [72,73]. Pyuria, being one of the commonest genitourinary manifestations, was reported in 30–80% of the KD patients with those below one-year-old being more likely to be affected [54]. TIN is typically the cause of acute kidney injury among patients with KD, which is the main pathogenesis due to abnormal T-cell activation, causing dysregulated immune responses in the renal tubules [74]. Urethritis is a common finding in KD, whereas hydrocoele and phimosis rarely occur [1]. In terms of renal abnormalities observed through imaging study, renal sonographic findings of increased cortical echogenicity, enlarged kidneys, and enhanced corticomedullary differentiation are common associations with KD, and are mostly due to vasculitis involving the kidney that causes cellular infiltration leading to ischemia and oedema [65].

### 4.4. Musculoskeletal System

KD also affected the musculoskeletal system with arthralgia and arthritis as the most prevalent conditions reported with KD [1]. Around 3% of patients in Southern Malaysia had musculoskeletal manifestations whereas 11% of patients with KD in China had arthritis [8,54]. Older children seem to be more likely to have arthritis when compared to those who are younger [54,70]. This is most probably due to a better understanding and communication skills among the older patients, who complain about their pain better than the younger patients [55]. Patients with arthritis have been associated with amplified risk of progressing toward a coronary artery aneurysm [54]. Several studies have found a small proportion (0.2–0.5%) of KD patients who subsequently developed systemic-onset juvenile idiopathic arthritis (sJIA) [75,76]. Interestingly, Kanemasa et al. has reported that KD-related arthritis involved both interval-type (KD-reactive) and continued-type (true sJIA) requiring biologics, may have a similar immunopathology with sJIA as there were overlapping clinical features between KD and sJIA [77]. There were cases where the KD patients have presented with myositis with a normal or mild elevation of the muscle enzymes, which causes proximal muscle weakness that requires the use of glucocorticoid as a treatment [78,79,80].

### 4.5. Respiratory System

The presence of cough, sputum production and rhinorrhoea are considered common symptoms in patients diagnosed with KD [55]. In a prospective study conducted in Taiwan, 69% of the KD patients had cough while 58% of these patients have subsequently developed rhinorrhoea [56]. In Korea, respiratory symptoms were also observed among KD patients as cough, rhinorrhoea and sputum production accounted for 46%, 31% and 15%, respectively [55]. The chest radiographs of patients with KD may reveal peribronchial and interstitial infiltrates, while nodular infiltrates were only seen in rare cases [1]. These findings resonate with He et al. [81] who found that peripheral consolidation was the predominant feature in the chest radiographs of KD patients. Umezawa et al. also mentioned that 129 acute KD patients manifested some abnormalities in the chest X-ray, with the most common finding being the reticulogranular pattern (89.5%), followed by peribronchial cuffing (21.1%), pleural effusion (15.8%), atelectasis (10.5%), and air trapping (5.3%) [82]. According to Lee et al., 51.8% of patients with KD from their cohort has concurrent *Mycoplasma pneumoniae* infection, evidenced by high titre of anti-M.pneumoniae antibody [83].These patients additionally presented with chest X-ray abnormalities, which include findings such as focal reticulonodular lesions, atelectasis and hilar lymphadenopathy [83]. Patients with pulmonary manifestations have a heightened risk of getting coronary artery abnormalities. This was possibly due to the delayed diagnosis of KD as the initial treatments were focusing on the misdiagnosis of pulmonary causes [84]. There are also other pulmonary manifestations observed in KD, including bronchopneumonia [85], hydropneumothorax [86] and pleural effusion [87]. In this regard, patients with pulmonary manifestations are often delayed in the diagnosis of KD as it is uncommon and atypical, thus physicians who encounter patients with difficult-to-treat bronchopneumonia should think of Kawasaki disease.

### 4.6. Neurological System

**Figure 1:**
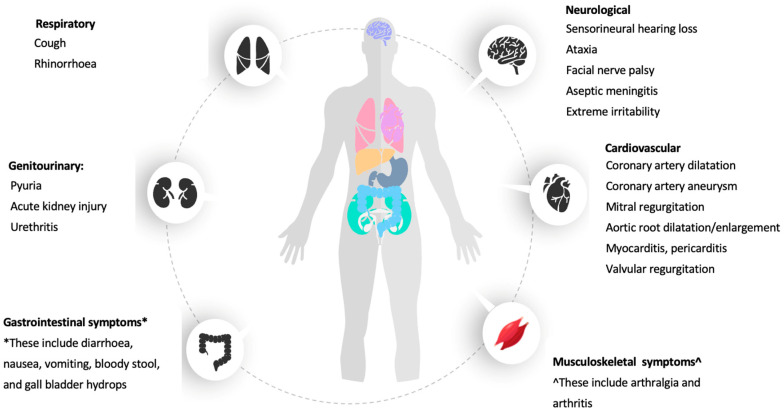
A summary of the clinical features of KD.

The neurological symptoms of KD include aseptic meningitis and severe irritability beyond that seen in other febrile diseases. Temporary unilateral peripheral facial nerve palsy (FNP) and sensorineural hearing loss (SNHL) had been observed in KD patients [1]. SNHL was reported as early as 1988 by Suzuki et al. from Japan, and 33–36% of children with KD were noted to present with some degree of SNHL after the disease onset [53,88]. In comparison, only approximately 2.8–5% of the KD patients were reported to develop neurological symptoms, including SNHL in Malaysia and Iran, probably due to the lack of awareness and screening of the neurological manifestations [17,89]. Given the increasingly reported incidences of SNHL, some studies have strongly urged the inclusion of audiology screening for patients with KD as SNHL can contribute to delayed development in cognitive and speech domains, if it is not diagnosed and remains untreated [88]. Unusual manifestations, such as ataxia and facial nerve palsy, had also been reported in KD patients [18,90,91]. Most of the patients who developed facial nerve palsy were below 20 months in age and IVIG treatments have shown to assist in its resolution [90]. Though rare, clinicians should be well-informed of FNP as an association with KD, as it was noted to be remarkably interrelated with an elevated risk of coronary artery lesions [90,91]. All the clinical features of KD are summarized in Figure 1.

## 5. Laboratory Characteristics of KD

To date, there is no specific biomarker that has been found to diagnose KD with a complete certainty. Although several potential biomarkers have been recently reported, none of these has been clinically validated and evaluated, and more importantly, accessible freely for a clinical usage. Nonetheless, several widely available and accessible laboratory tests can be performed to assist clinicians in diagnosing KD, which include full blood count, erythrocyte sedimentation rate (ESR), and C-Reactive Protein (CRP), liver function test (LFT) and renal profile (RP), among others. Even though these tests do not serve as the diagnostic investigation for KD, they are most readily available and can help to detect the severity of other organs’ involvements and for supportive management. In this section, we will review the abnormalities observed in KD in the routinely available and accessible investigations in most healthcare facilities.

### 5.1. Full Blood Count and Inflammatory Markers (CRP, ESR, PCT, Serum Ferritin, D-Dimer/Fibrinogen Level)

Normochromic normocytic anaemia is a common abnormality observed in KD patients and this abnormality disappears when inflammation attenuates [1]. Findings from a retrospective study by Malik et al. [17] have revealed that anaemia, thrombocytosis, and high ESR were the most frequent reported findings among KD patients. This is parallel to the latest study which also revealed that 49.3% of the enrolled KD patients had thrombocytosis and it was strongly linked to a poorer prognosis such as IVIG unresponsiveness [92]. Acute phase reactants, especially ESR and CRP, are raised and leukocytosis is commonly seen in acute KD [93]. On average, elevated ESR level was seen in most of the patients, followed by thrombocytosis, leukocytosis, raised CRP and anaemia. Thus, KD can be excluded if the ESR, CRP and platelet counts remain within the normal range after the seventh day of the disease [1]. The comparison of full blood count abnormalities in different findings are summarized in Table 2.

Other inflammatory markers that often raised in the acute phase of KDare ferritin, D-dimer, fibrin degradation products and procalcitonin. In comparison to the IVIG responder group of KD patients, the IVIG resistant group had significantly higher D-dimer and serum ferritin levels [97,98,99,100]. The D-dimer level had a significant sensitivity of 87.0% and a specificity of 56.3% in predicting the IVIG resistance at a cut-off point of 1.09 mg/L [100]. Apart from that, the serum ferritin level was noted to have a sensitivity of 42.9% and a specificity of 88.8% for predicting IVIG resistance at a cut-off point of 269.7 ng/mL [100]. The D-dimer level also reported a sensitivity of 50% and a specificity of 78.6% in presuming coronary artery lesions at a cut-off point of 1.84 mg/L [100]. However, a study also revealed a moderate sensitivity (57.10%) and a high specificity (82.90%) of serum ferritin in predicting IVIG resistance [99]. On the contrary, it had a sensitivity level of 92.30% and a specificity level of 37.70% in predicting coronary artery lesions [99].

However, the increased D-dimer levels had no association with coronary artery lesions [101]. KD patients with elevated D-dimer have a higher incidence of granulopenia, thrombocytosis, myocardial damage, cholestasis, hypoproteinemia, and aseptic urethritis when compared to those with normal levels of D-dimer [101]. The increased D-dimer and fibrin degradation product values would guide the diagnosis of incomplete KD. However, they are unable to predict any cardiac complications in KD patients [101]. This study found a cut-off value of 1.1 μg/mL for D-dimer to diagnose KD with a sensitivity of 0.95 and specificity of 0.59 [101]. Similarly, a cut-off value for fibrin degradation product was set as 2.5 μg/mL with a sensitivity of 0.89 and a specificity of 0.70 [101].

The increased level of procalcitonin was seen among KD patients in the acute phase, especially in those with resistance to IVIG [102,103,104]. A cut-off value of procalcitonin (0.5 ng/mL) for IVIG resistance signifies a sensitivity of 85% and the accuracy of 64% [102,104]. Altogether, is it suggested that KD patients with procalcitonin levels of more than 0.5 ng/mL had a notably greater incidence of IVIG resistance.

### 5.2. Liver Function Test and Lipid Profile

Patients with acute KD are commonly presented with a deranged liver function test (LFT). A study has reported that 37% of the patients with KD had a significant rise in the levels of alanine aminotransferase (ALT) and aspartate aminotransferase (AST) while 41% of them had an elevated gamma-glutamyl transferase (GGT) level [105]. Similarly, another study also reported that most children with KD had impaired LFT with raised ALT and/or AST [106]. In a retrospective review that scrutinised the liver function profile among 210 KD children, hypoalbuminemia was prevalent along with high AST and low total protein during the acute KD period [17]. Elevated AST and ALT, as well as hypoalbuminaemia, are linked with IVIG resistance and coronary artery aneurysm [105,107]. From a similar angle, it was noted that patients with a high AST: ALT ratio has an increased risk of getting coronary artery lesion in KD [108]. It had been suggested that deranged liver parameters are the possible manifestations of atypical KD, thus signifying the importance of performing liver function tests in patients suspected of KD to assist in making an earlier diagnosis [109]. Besides the level of AST and ALT, the GGT level has also emerged as one of the independent predictors of diagnosing KD. A study has found that almost half of the KD patients (5034 out of 10,367 patients) have a raised GGT level, in addition to the increased level of ALT [110]. High levels of AST, ALT and GGT, alongwith the low levels of albumin, could indicate a severe inflammation in the liver which may correlate to the severity of the inflammatory changes in the vessels and other organs [111].

In addition to the liver function test, lipid profile may serve as another tool to look at abnormal parameters, especially in the acute phase of KD. Lipid profiles among 105 KD patients within three years of the diagnosis have noted a decreasing trend in total cholesterol and HDL levels, and an elevated TG level [112,113]. These changes might lead to premature atherosclerosis, hence monitoring of the lipid profile is important in KD patients [114]. The HDL level is inversely correlated with the CRP level in predicting coronary artery lesions. It was noticed that KD patients with coronary artery lesions had higher levels of CRP and low HDL levels while this feature was not observed in KD patients without coronary artery lesions, making, these combined CRP and HDL levels a candidate predictor for the development of coronary artery lesion (CAL) in KD [115]. Additionally, it was also noted that there is a positive relation between CRP level and the size of CAL, nonetheless, this association was not observed between HDL level and the CAL size [115]. The inconclusive data may warrant a further study to further investigate the role of lipid profile in correlation to KD.

### 5.3. Renal Function and Urinary Sample Assessment

Patients with KD may develop proteinuria, microscopic hematuria, sterile pyuria, and glycosuria during the acute phase of KD [116,117], with pyuria being the most common feature [72]. 28% of KD patients below two years old had developed acute kidney injury (AKI) with their serum creatinine level raised more than two-fold the normal upper limit [118]. In the same cohort of patients, sterile pyuria was observed in 31.5% of the patients and it was also noted to be most prevalent in KD patients younger than one-year-old [118]. There have been reported cases that those with bladder and urethral pyuria have higher serum blood urea nitrogen (BUN) and creatinine levels when compared to those with non-pyuria [53]. Nonetheless, pyuria is not necessarily sterile in KD patients as it can be due to concurrent urinary tract infection [53]. Hence, mid-stream urine culture and sensitivity need to be performed and the most common organisms concurrently causing urinary tract infection in KD are Escherichia coli and Klebsiella oxytoca [53]. UTI can also be differentiated from KD pyuria by the positive nitrite test, as it is more specific for the diagnosis of UTI [119]. Additionally, hyponatremia was observed in 45% of KD patients and it was linked to a more serious inflammatory event such as a longer febrile period [120].

### 5.4. Autoimmune and Cardiovascular Biomarkers

As a known systemic vasculitis affecting the vessels, KD was reported to cause proinflammatory hypercytokinemia, which subsequently may precede the occurrence of coronary artery lesions [121,122]. Several evidence of the development of self-antigens in response to inflammation such as anti-endothelial cells antibodies (AECA) and anti-neutrophil cytoplasmic antibodies (ANCA) in the course of KD vasculitis, suggest a possible role of these antibodies as indicators for early diagnosis of KD in the future [123]. Activation of B cells and cytotoxic T cells, endothelial damage with thrombin formation was noted to occur in the acute phase of KD, further indicating that the autoimmune component plays a part in the pathogenesis of KD [122]. However, none of these markers had been reported in the large cohort of KD patients.

Several cardiovascular biomarkers have shown to increase in KD patients but none of them is specific since the presence of these markers generally indicates myocardial injury. N-terminal prohormone Brain Natriuretic Peptide (NT-proBNP) is the most studied marker in KD due to its usefulness in diagnosis and prognostic value [123]. Raised NT-proBNP correlates with the manifestation of coronary artery aneurysm and can predict the presence of IVIG resistance in KD patients [123,124]. Furthermore, the usage of NT-proBNP as a diagnostic marker was also substantiated by multiple studies and meta-analyses [125,126,127,128]. Although NT-proBNP is non-specific andlimiting the diagnostic usefulness of this marker for KD, the usefulness warrants future studies to investigate the value of NT-proBNP in either diagnostic, prognostic, or treatment algorithms with the combination of other clinical criteria and laboratory findings [123]. Besides that, serum BNP level also showed a negative correlation with cardiac functions in KD patients such as lower left ventricular ejection fraction, reduced left ventricular systolic function and cardiac index in KD [125].

Other than that, Cardiac Troponin I (cTnI) is shown to be elevated along with CK-MB in acute KD; however, both markers are not specific and lack usefulness in the management of KD [128]. Another study has shown that elevation of cTnI was insignificant in KD patients and provided no correlation with the development of CAA or myocarditis [127]. Other cardiac biomarkers, such as plasma soluble suppression of tumorigenesis-2 (sST2) along with Gamma Glutamyl Transferase (GGT) and alanine transaminase (ALT) are also significantly raised in acute KD [128]. However, sST2 is rarely used in a clinical setting while GGT and ALT elevation is not specific to cardiac involvement. As a whole, the presence of serum cardiovascular markers indicates myocardial inflammation in KD, which may reflect the underlying cardiomyocyte stress and cell death [128].

## 6. Conclusions

KD is a well-recognised autoinflammatory paediatric disorder, and it has been widely reported worldwide particularly in Asia in the last few decades. Apart from the five diagnostic classic features, KD also involves other different body organ systems including the cardiovascular, gastrointestinal, neurological, and musculoskeletal systems. These manifestations can be further utilised to develop a more thorough and comprehensive guideline in assisting to diagnose KD and further delineate the separation of KD and other diseases mirroring KD clinical manifestations.

Additionally, we also reviewed the abnormalities commonly observed in patients with KD, based on the widely accessible list of investigations available in most healthcare facilities. Although at this moment, the laboratory findings can only be used to reinforce the diagnosis of KD, they serve as important tools for the healthcare providers to prognosticate the treatment like IVIG resistance and assess the severity of the disease for prompt treatment to be implemented. In addition to what had been presented, ongoing studies are required to identify other unknown or “yet to be described” specific manifestations of KD, so that healthcare providers may have a better clinical suspicion when encountering children with KD. As this matter is highly crucial, it is of global interest and benefit to mankind for the correct, prompt, and earlier diagnosis to be found and applied, so that the appropriate treatment can be implemented to reduce the probability of co-morbidities and other complications.

## Figures and Tables

**Table 1:** Summary findings of cardiac manifestation from different studies *.

Authors	Saundankar et al. (2014) [28]	Sanchez-Manubens et al. (2014) [58]	Ae et al. (2021) [59]	Pinto et al. (2017) [60]	G. B. Kim et al. (2017) [61]	Xie et al. (2020) [62]	Son et al. (2020) [63]
Country (*n* sample)	Australia *n* = 281	Spain *n* = 398	Japan *n* = 3714	Italy *n* = 470	South Korea *n* = 12,269	China *n* = 4442	Vietnam *n* = 167
Coronary artery dilation/ecstasia #, *n* (%)	47 (16.7)	N/A	3343 (90)	N/A	1328 (10.8)	231 (5.2)	N/A
Coronary artery aneurysm/small aneurysm #, *n* (%)	14 (5.0)	53 (13.3)	316 (9)	40 (8.5)	207 (1.7)	N/A	49 (29.2)
Medium aneurysm, *n* (%)	N/A	N/A	N/A	N/A	N/A	118 (2.7)	60 (9.6)
Large/giant aneurysm, *n* (%)	5 (1.8)	N/A	49 (1)	N/A	19 (0.16)	31 (0.7)	N/A
Myocarditis, *n* (%)	N/A	4 (1)	N/A	N/A	N/A	N/A	N/A
Pericarditis, *n* (%)	N/A	9 (2.3)	N/A	18 (3.8)	N/A	N/A	N/A
Valvular lesion, *n* (%)	N/A	28 (7)	N/A	2 (0.4)	N/A	653 (14.7)	2 (1.2)

N/A: Not Available. * Cardiovascular findings include those diagnosed during hospitalisation and follow up. # Coronary artery aneurysm and dilatation were determined in accordance with the Japanese Ministry of Health (JCS Joint Working Group, 2014; Research Committee on Kawasaki Disease, 1984).

**Table 2:** Comparison of Full Blood Count (FBC) abnormalities of different studies in KD (selected based on the FBC variables).

FBC Variables	Malik et al. (1996) [16] *n* = 7	Xuan et al. (2020) [63] *n* = 168	Singh et al. (2005) [94] *n* = 69	Zhu et al. (2015) [95] *n* = 226	Ghandi et al. (2020) [96] *n* = 69	Mean (%)
Anaemia, *n* (%)	4 (57.1)	147 (87.5)	N/A	89 (39.4)	17 (24.6)	52.2
Leukocytosis, *n* (%)	6 (85.7)	142 (84.5)	39 (56.5)	138 (61.1)	24 (34.8)	64.5
Thrombocytosis, *n* (%)	5 (71.4)	58 (34.5)	36 (52.2)	208 (92.0.)	55 (79.7)	66.0
High Erythrocyte Sedimentation Rate (ESR), *n* (%)	5 (71.4)	94 (54.6)	37 (75.5)	172 (79.3)	54 (78.3)	71.8
High C-Reactive Protein (CRP), *n* (%)	N/A	128 (76.2)	28 (62.2)	133 (61.6)	20 (29.0)	57.3

N/A: Not Available.

## Data Availability

Not applicable.
